# COVID-19 Testing to Sustain In-Person Instruction and Extracurricular Activities in High Schools — Utah, November 2020–March 2021

**DOI:** 10.15585/mmwr.mm7021e2

**Published:** 2021-05-28

**Authors:** William A. Lanier, Kendra D. Babitz, Abigail Collingwood, Maggie F. Graul, Sydnee Dickson, Lexi Cunningham, Angela C. Dunn, Duncan MacKellar, Adam L. Hersh

**Affiliations:** ^1^Utah Department of Health; ^2^Utah State Board of Education; ^3^Utah School Superintendents Association, Sandy, Utah; ^4^CDC COVID-19 Response Team; ^5^Department of Pediatrics, Division of Infectious Diseases, University of Utah, Salt Lake City, Utah.

Cessation of kindergarten through grade 12 in-person instruction and extracurricular activities, which has often occurred during the COVID-19 pandemic, can have negative social, emotional, and educational consequences for children (*1*,*2*). Although preventive measures such as masking, physical distancing, hand hygiene, and improved ventilation are commonly used in schools to reduce transmission of SARS-CoV-2, the virus that causes COVID-19, and support in-person instruction (*3*–*6*), routine school-based COVID-19 testing has not been as widely implemented. In addition to these types of standard preventive measures, Utah health and school partners implemented two high school testing programs to sustain extracurricular activities and in-person instruction and help identify SARS-CoV-2 infections: 1) Test to Play,* in which testing every 14 days was mandated for participation in extracurricular activities; and 2) Test to Stay,^†^ which involved school-wide testing to continue in-person instruction as an alternative to transitioning to remote instruction if a school crossed a defined outbreak threshold (*3*). During November 30, 2020–March 20, 2021, among 59,552 students tested through these programs, 1,886 (3.2%) received a positive result. Test to Play was implemented at 127 (66%) of Utah’s 193 public high schools and facilitated completion of approximately 95% of scheduled high school extracurricular winter athletics competition events.^§^ Test to Stay was conducted at 13 high schools, saving an estimated 109,752 in-person instruction student-days.^¶^ School-based COVID-19 testing should be considered as part of a comprehensive prevention strategy to help identify SARS-CoV-2 infections in schools and sustain in-person instruction and extracurricular activities.

For both the Test to Play and Test to Stay programs, the Utah Department of Health (UDOH) provided training and rapid antigen test kits** to school staff members, who performed school-based rapid antigen testing (e.g., in school gymnasiums), supported by UDOH and local health departments. Parental permission was required for students to receive school-based testing. Schools were required to report all test results to UDOH. In lieu of school-based testing, students could participate in these programs by receiving testing elsewhere (e.g., via community testing). Students who had a negative test result were allowed to continue to participate in in-person instruction and extracurricular activities; students who had a positive test result were required to isolate for 10 days from the date of the test, and close contacts were required to quarantine^††^ (*3*). For Test to Stay events, schools were advised that students who opted out of testing should transition to remote instruction for 10 days from the date of event.

The UDOH COVID-19 surveillance system was used to evaluate trends in COVID-19 incidence among children aged 5–17 years and Test to Play and Test to Stay results.^§§^ In addition, UDOH administered a survey to school representatives in February 2021 to identify facilitators of and barriers to conducting Test to Stay and to collect information on testing events. In March 2021, UDOH also collected data from all school districts on outbreak threshold crossings and transitions to remote instruction. These activities were reviewed by CDC and were conducted consistent with applicable federal law and CDC policy.^¶¶^

Beginning August 2020, 40 of 41 Utah school districts opened for in-person instruction.*** In September 2020, COVID-19 incidence in Utah among persons aged 14–17 years rose rapidly, followed by similar but smaller increases among persons aged 5–13 years (Figure 1). On November 9, statewide COVID-19 restrictions were ordered, including a cessation of extracurricular activities except high school football.^†††^ In mid-November, Test to Play was piloted among participants in high school football state championships. Beginning November 30, Test to Play was mandated for participants in all high school extracurricular activities (Figure 2).

**FIGURE 1:**
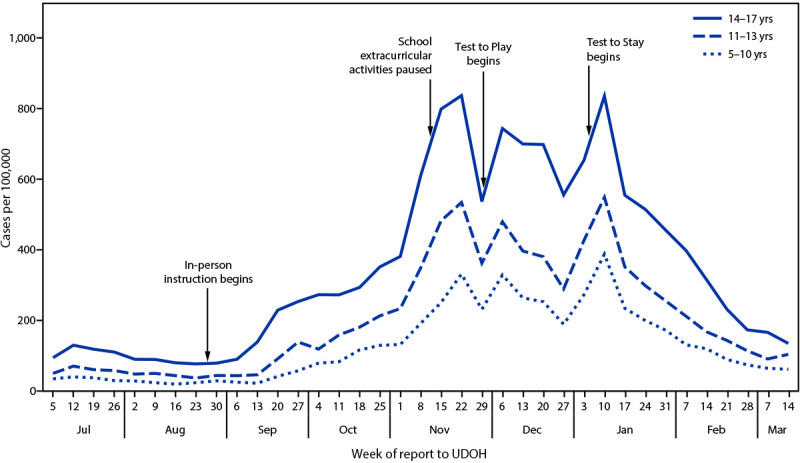
COVID-19 incidence* among children aged 5–10 years (N = 311,812), 11–13 years (N = 161,991), and 14–17 years (N = 209,578), by week — Utah, July 5, 2020–March 14, 2021^†^^,^^§^^,^^¶^^,^** **Abbreviation:** UDOH = Utah Department of Health. * Total new cases per 100,000 persons in the previous 7 days, calculated using 2018 population data. https://ibis.health.utah.gov ^†^ In August 2020, Utah schools opened for in-person instruction in 40 of 41 school districts. ^§^ On November 9, 2020, Utah State Public Health Order 2020-21 limited participation in organized extracurricular activities to high school football practice or games. https://coronavirus-download.utah.gov/Health/UPHO-2020-21-Temporary-Statewide-COVID-19-Restrictions.pdf, https://coronavirus-download.utah.gov/School/COVID-19_School_Manual_FINAL.pdf ^¶^ Test to Play, which required testing every 14 days for participants in high school extracurricular activities, is described in the Utah COVID-19 School Manual (https://coronavirus-download.utah.gov/School/COVID-19_School_Manual_FINAL.pdf) and was mandated by Utah State Public Health Order 2020-25 (https://coronavirus-download.utah.gov/Health/UPHO_2020-25_Statewide_COVID-19_Restrictions.pdf), effective November 30, 2020. ** Beginning August 2020, schools were advised to transition to remote instruction for 14 days when the number of school-associated cases among students and staff members crossed a specified outbreak threshold. During August–December 2020, the outbreak threshold was 15 school-associated cases during the previous 14 days. Under Test to Stay (https://coronavirus-download.utah.gov/School/COVID-19_School_Manual_FINAL.pdf), which began January 4, 2021, the outbreak threshold of cases during the previous 14 days changed to 1% of the school population for schools with >1,500 students and staff members and 15 cases for schools with ≤1,500 students and staff members, and the period of advised remote instruction after crossing the outbreak threshold changed to 10 days.

**FIGURE 2:**
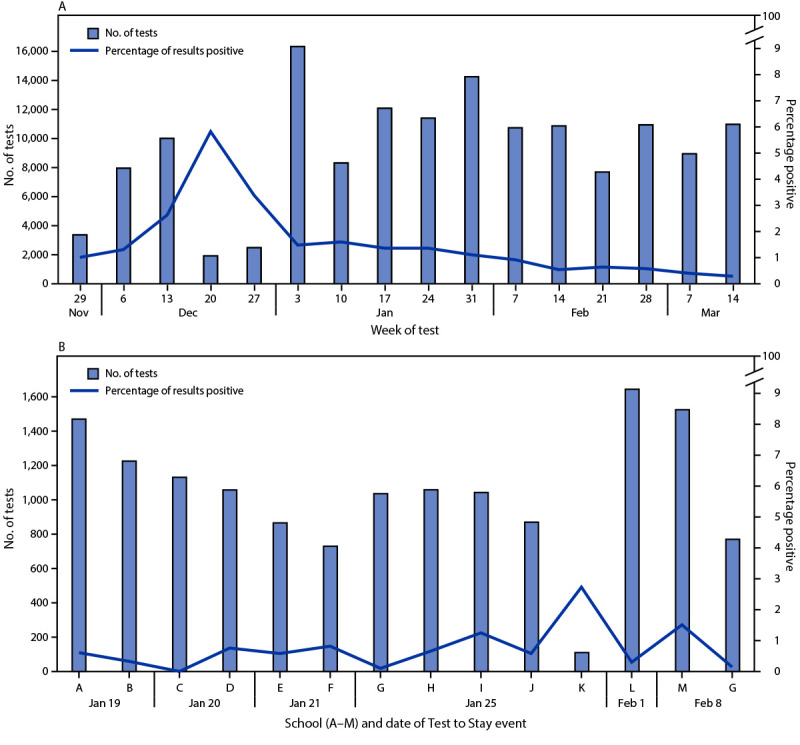
Number of school-based SARS-CoV-2 rapid antigen tests* performed and percentage positive among students participating in Test to Play (A)^†^ and Test to Stay (B),^§^ by week or date of testing event and high school (for Test to Stay) — Utah, November 30, 2020–March 20, 2021 * Abbott BinaxNOW rapid antigen nasal swab test kits were provided at no cost to the Utah Department of Health by the U.S. Department of Health and Human Services. https://www.fda.gov/media/141570/download ^†^ Test to Play, which required testing every 14 days for participants in high school extracurricular activities, is described in the Utah COVID-19 School Manual (https://coronavirus-download.utah.gov/School/COVID-19_School_Manual_FINAL.pdf) and was mandated by Utah State Public Health Order 2020-25 (https://coronavirus-download.utah.gov/Health/UPHO_2020-25_Statewide_COVID-19_Restrictions.pdf), effective November 30, 2020. ^§^ Beginning August 2020, schools were advised to transition to remote instruction for 14 days when the number of school-associated cases among students and staff members crossed a specified outbreak threshold. During August–December 2020, the outbreak threshold was 15 school-associated cases during the previous 14 days. Under Test to Stay (https://coronavirus-download.utah.gov/School/COVID-19_School_Manual_FINAL.pdf), which began January 4, 2021, the outbreak threshold of cases during the previous 14 days changed to 1% of the school population for schools with >1,500 students and staff members and 15 cases for schools with ≤1,500 students and staff members, and the period of advised remote instruction after crossing the outbreak threshold changed to 10 days. Each of the 13 schools that conducted Test to Stay is represented by a unique letter (A–M). With the exception of the second high school G event, Test to Stay events were conducted over 1 or 2 days and all student testing reported by these schools during the events was counted as Test to Stay. Multiday Test to Stay events are indicated on the first day of the event. High school G conducted a Test to Stay event during January 25–26 after crossing the outbreak threshold. Subsequently, high school G conducted modified, follow-up Test to Stay testing during February 8–March 9; all Test to Stay testing among students reported by this school during this period is represented as a single, additional event. High school K conducted a Test to Stay event when the school was approaching, but had not reached, the outbreak threshold.

During August–December 2020, schools crossing the defined outbreak threshold were recommended to transition temporarily to remote instruction, in consultation with their local health departments. During this period, Utah school districts reported 78 high school transitions to remote instruction after crossing the outbreak threshold. In December 2020, Test to Stay was piloted at two high schools. Beginning January 4, 2021, schools crossing the outbreak threshold could choose to implement Test to Stay as an alternative to transitioning to remote instruction (Figure 2).

During November 30, 2020–March 20, 2021, a total of 165,078 tests among high school students were reported in Test to Play and Test to Stay. Among 59,552 students receiving testing at least once, including one third (34%) of Utah’s public high school students, 1,886 (3.2%) had a positive result.^§§§^

During the same period, public and private schools and school districts, including 127 (66%) of Utah’s 193 public high schools, reported 148,262 Test to Play tests among high school students.^¶¶¶^ Among 50,400 students receiving testing at least once, representing an estimated two thirds (67%) of all high school students participating in extracurricular activities, 1,771 (3.5%) had a positive result.**** During January 3–March 20, 2021, the percentage of positive tests declined (Figure 2), consistent with decreasing statewide incidence among school-aged children during this period (Figure 1). Test to Play allowed extracurricular activities to occur in the context of mandated testing; during November 30, 2020–February 20, 2021, approximately 95% of the 11,379 scheduled competition events for high school extracurricular winter athletics were completed.

School districts reported 29 outbreak threshold crossings in 28 high schools during January 4–March 5, 2021; 16 of these schools chose not to conduct Test to Stay and transitioned to remote instruction. During January 4–March 20, 2021, 13 high schools conducted 14 Test to Stay events, performing 14,531 tests among students (Figure 2). Among 13,809 students receiving testing at least once during these events, representing an estimated 70% of students participating in in-person instruction at these 13 schools,^††††^ 90 (0.7%) had a positive result (range of test positivity among events = 0.0%–2.7%). After testing events, these 13 schools continued in-person instruction, collectively saving an estimated 109,752 in-person instruction student-days.

Among the 303 Utah public and private schools and school districts included in the UDOH survey, representatives from 144 (48%) responded. Identified facilitators of Test to Stay included promoting student participation through pre-event parental messaging and preregistration for testing, coordinating with health partners to increase testing capacity, and maintaining in-person instruction during testing. Barriers included lack of perceived community support and limited staffing capacity (Box).

BOX Important facilitators of and barriers to conducting Test to Stay* — survey of Utah schools and school districts, February 2021^†^

**Facilitators**

- Early planning and staff member training before a school reaches the outbreak threshold to maximize preparedness
- Delivery of messaging to students and parents or guardians before the event to promote student participation
- Encouraging students to preregister to save time during the event
- Leveraging existing school capacity for testing, such as Test to Play^§^
- Coordination with local and state health departments to increase testing capacity and facilitate a coordinated event
- Maintaining in-person instruction during the event

**Barriers**

- Lack of perceived support for student testing among school boards, student families, or community members
- Limited staffing capacity for large-scale testing
- Concern among teachers that testing will lead to dual-modality instruction (both in-person and virtual), particularly if low numbers of students participate in testing
- Difficulty reporting test results due to unreliable Internet access, school security firewalls, or user errors when generating registration links
- Mistaken belief that a school would be ineligible for Test to Stay if it did not participate in Test to Play

* Beginning August 2020, schools were advised to transition to remote instruction for 14 days when the number of school-associated cases among students and staff members crossed a specified outbreak threshold. During August–December 2020, the outbreak threshold was 15 school-associated cases during the previous 14 days. Under Test to Stay (https://coronavirus-download.utah.gov/School/COVID-19_School_Manual_FINAL.pdf), which began January 4, 2021, the outbreak threshold of cases during the previous 14 days changed to 1% of the school population for schools with >1,500 students and staff members and 15 cases for schools with ≤1,500 students and staff members, and the period of advised remote instruction after crossing the outbreak threshold changed to 10 days.

^†^ The Utah Department of Health administered a survey of school representatives to determine facilitators of and barriers to conducting Test to Stay. Of 303 Utah public and private schools and school districts included in the survey, representatives from 144 (48%) responded.

^§^ Test to Play, which required testing every 14 days for participants in high school extracurricular activities, is described in the Utah COVID-19 School Manual (https://coronavirus-download.utah.gov/School/COVID-19_School_Manual_FINAL.pdf) and was mandated by Utah State Public Health Order 2020–25 (https://coronavirus-download.utah.gov/Health/UPHO_2020-25_Statewide_COVID-19_Restrictions.pdf), effective November 30, 2020.

## Discussion

Utah’s high school COVID-19 testing programs saved in-person instruction days and facilitated continuation of extracurricular activities in accordance with statewide public health policy during a period of high COVID-19 incidence among persons of high-school student age. Growing evidence suggests that when schools implement recommended prevention strategies, including consistent and correct use of masks, physical distancing, hand hygiene, and room ventilation improvements, in-school COVID-19 transmission is infrequent (*4*,*5*,*7*), while loss of in-person instruction can have detrimental effects on children’s education and their social and emotional well-being (*1*,*2*). Consistent and correct mask use remains recommended by CDC for adults and children in schools, regardless of vaccination status.^§§§§^ Outcomes of Utah’s Test to Play and Test to Stay programs are consistent with those from a screening program implemented in a New Jersey boarding school (*6*), suggesting that school-based COVID-19 screening can be a feasible component of a comprehensive, multicomponent prevention approach (*3*) that helps sustain in-person instruction and extracurricular activities.

By identifying 1,886 cases among students, Utah’s testing programs likely helped reduce SARS-CoV-2 transmission in schools and communities through isolation of students with diagnosed infections and quarantine of contacts. Routine Test to Play testing also provided complementary community surveillance for SARS-CoV-2 infection among high school students, many of whom were likely not experiencing symptoms that would have prompted testing and diagnosis elsewhere. In addition, linking serial testing results to socially desirable activities, such as participation in extracurricular activities, might have incentivized masking and other preventive behaviors.

Although many cases of SARS-CoV-2 infection were diagnosed in the Test to Play and Test to Stay rapid antigen testing programs, strategies using more sensitive nucleic acid amplification tests would likely detect more cases (*6*,*8*,*9*). Rapid antigen testing, however, is less expensive, provides results in 15 minutes, and avoids burdening laboratories. Even though screening more than once every 2 weeks or at a lower outbreak threshold could also detect more cases, frequent rapid antigen testing in the context of low prevalence (<1.0%) would likely produce excess false-positive SARS-CoV-2 results at a high cost (*10*).^¶¶¶¶^ Utah’s school-based testing programs were implemented using rapid antigen testing according to the parameters described in this report to balance resources and feasibility with test performance, and to enable timely isolation, investigation of cases, and quarantine of contacts.

Notably, even with the provision of free test kits, training, and testing assistance, fewer than one half of schools that crossed the outbreak threshold chose to sustain in-person instruction by implementing Test to Stay. To help overcome identified barriers to implementing Test to Stay, UDOH, with health and education partners, continues to provide community messaging materials and additional staffing to support testing events.

The findings in this study are subject to at least three limitations. First, test numbers are underestimated because all testing in these programs might not have been reported and results of testing performed separately from school-based testing (e.g., via community testing) were not classified as Test to Play or Test to Stay. Second, these testing programs did not include collection of data on the clinical status and isolation of students with diagnosed SARS-CoV-2 infection, number of close contacts identified and quarantined, or exposure settings. Finally, the impact of these testing programs or other interventions (e.g., masking) on COVID-19 transmission in schools was not assessed.

Because interruption of in-person instruction and extracurricular activities can negatively affect children, strategies that safely facilitate student participation in these activities are important. Additional research is needed to determine the optimal operational parameters for school-based COVID-19 screening, including testing frequency, outbreak threshold, and the role of screening in the context of vaccination. Utah’s approach could serve as a framework for other jurisdictions considering school-based testing as part of a comprehensive prevention strategy to help identify SARS-CoV-2 infections while sustaining in-person instruction and extracurricular activities.

**Summary**

**What is already known about this topic?**

COVID-19–associated cessation of kindergarten through grade 12 in-person instruction and extracurricular activities can have negative social, emotional, and educational consequences for children.

**What is added by this report?**

Utah implemented two high school COVID-19 testing programs to sustain in-person instruction and extracurricular activities. During November 30, 2020–March 20, 2021, among 59,552 students who received testing, 1,886 (3.2%) had a positive result. These programs facilitated the completion of approximately 95% of high school extracurricular competition events and saved an estimated 109,752 in-person instruction student-days.

**What are the implications for public health practice?**

School-based COVID-19 testing should be considered part of a comprehensive prevention strategy to identify SARS-CoV-2 infections in schools and sustain in-person instruction and extracurricular activities.

## Footnotes

* Test to Play, which required testing every 14 days for participants in high school extracurricular activities, is described in the Utah COVID-19 School Manual (https://coronavirus-download.utah.gov/School/COVID-19_School_Manual_FINAL.pdf) and was mandated by Utah State Public Health Order 2020-25 (https://coronavirus-download.utah.gov/Health/UPHO_2020-25_Statewide_COVID-19_Restrictions.pdf), effective November 30, 2020.

† Beginning August 2020, schools were advised to transition to remote instruction for 14 days when the number of school-associated cases among students and staff members crossed a specified outbreak threshold. During August–December 2020, the outbreak threshold was 15 school-associated cases during the previous 14 days. Under Test to Stay (https://coronavirus-download.utah.gov/School/COVID-19_School_Manual_FINAL.pdf), which began January 4, 2021, the outbreak threshold of cases during the previous 14 days changed to 1% of the school population for schools with >1,500 students and staff members and 15 cases for schools with ≤1,500 students and staff members, and the period of advised remote instruction after crossing the outbreak threshold changed to 10 days. Beginning March 24, 2021 (after the study period), per Utah Senate Bill 107 (https://le.utah.gov/~2021/bills/static/SB0107.html), the outbreak threshold changed again, such that a school would be required to conduct Test to Stay if student cases during the previous 14 days reached 2% of the school’s student population for schools with ≥1,500 students and 30 students for schools with <1,500 students.

§ Of the 11,379 competition events scheduled for winter athletics sanctioned by the Utah High School Activities Association during November 30, 2020–February 20, 2021, a total of 10,812 (95%) occurred, including approximately 861 after being rescheduled. The Utah High School Activities Association recommended COVID-19 preventive behaviors during these extracurricular activities, including mask use and physical distancing. https://www.uhsaa.org/sportsmedicine/UHSAA%20Return%20to%20Play%20Document%20Winter%20Sport-by-Sport%20USSA%20UHSAA%20Plan.pdf

¶ Assumes that an average of 8 learning days were lost during each school transition to remote learning and that all 13,719 students who received negative test results during Test to Stay events at these 13 schools continued to participate in in-person instruction.

** Abbott BinaxNOW rapid antigen nasal swab test kits were provided at no cost to UDOH by the U.S. Department of Health and Human Services. https://www.fda.gov/media/141570/download

†† The required quarantine period for persons in close contact with an infected person changed from 14 days to 10 days beginning December 4, 2020.

§§ Persons aged 14–18 years whose Test to Play or Test to Stay test results were reported from a public high school, school district, or private school were considered to be high school students.

¶¶ 45 C.F.R. part 46, 21 C.F.R. part 56; 42 U.S.C. Sect. 241(d); 5 U.S.C. Sect. 552a; 44 U.S.C. Sect. 3501 et seq.

*** Each Utah local education authority could choose whether to open for in-person instruction for fall 2020. Among the 40 school districts that chose to open for in-person instruction, 39 began in August 2020, and one began in September 2020. One school district offered only remote instruction during fall 2020 and early 2021 and opened for in-person instruction in February 2021.

††† Utah State Public Health Order 2020-21 (https://coronavirus-download.utah.gov/Health/UPHO-2020-21-Temporary-Statewide-COVID-19-Restrictions.pdf). Because the order occurred at the end of its season, high school football was excepted to allow completion of state championship games.

§§§ Includes data from the December 2020 Test to Stay pilot, in which 2,285 students received testing and 26 (1.1%) had a positive result. Of 59,552 students tested in Test to Play and Test to Stay, 6,940 received testing in both programs, including two who received testing at two different Test to Stay events and one who had a positive result in both Test to Play and Test to Stay. In addition to those performed among students, 22,374 tests were performed among school staff members; of 9,688 school staff members who received testing at least once, 693 (7.2%) had a positive result. Of 59,552 students who received testing, 58,373 were public school students, representing 34% of the 172,260 students enrolled in Utah public high schools according to Utah State Board of Education fall 2020 enrollment data.

¶¶¶ High schools from which UDOH did not receive a report of Test to Play testing likely did not offer extracurricular activities, offered testing to extracurricular activity participants but did not report such testing, reported it under the school district rather than the school, reported it in a manner not identifiable as Test to Play, or required extracurricular activity participants to receive testing separately from school-based testing (e.g., community testing). The number of public high schools in Utah was supplied by the Utah State Board of Education.

**** An estimated 75,510 high school students participated in extracurricular activities during late November 2020–early March 2021 according to the Utah High School Activities Association. The 67% Test to Play participation estimate does not account for possible underreporting of school-based student testing or those students who might have received testing separately from school-based testing (e.g., via community testing).

†††† According to fall 2020 high school enrollment data reported by the Utah State Board of Education and school district–level proportions of high school students participating in full-time remote instruction reported by school districts, an estimated 19,660 students were participating in in-person instruction at these 13 high schools. The 70% Test to Stay participation estimate does not account for possible underreporting of school-based student testing, students who might have received testing separately from school-based testing (e.g., via community testing), or those not eligible for testing (e.g., a person who had a positive COVID-19 test result <90 days before the testing event).

§§§§ https://www.cdc.gov/coronavirus/2019-ncov/community/schools-childcare/k-12-testing.html, https://www.cdc.gov/coronavirus/2019-ncov/community/schools-childcare/operation-strategy.html#testing

¶¶¶¶
https://www.fda.gov/medical-devices/letters-health-care-providers/potential-false-positive-results-antigen-tests-rapid-detection-sars-cov-2-letter-clinical-laboratory

## References

[1] Van Lancker W, Parolin Z. COVID-19, school closures, and child poverty: a social crisis in the making. Lancet Public Health 2020;5:e243–4. 10.1016/S2468-2667(20)30084-0 32275858 PMC7141480

[2] Verlenden JV, Pampati S, Rasberry CN, Association of children’s mode of school instruction with child and parent experiences and well-being during the COVID-19 pandemic—COVID Experiences Survey, United States, October 8–November 13, 2020. MMWR Morb Mortal Wkly Rep 2021;70:369–76. 10.15585/mmwr.mm7011a1 33735164 PMC7976614

[3] Utah Department of Health, Utah Association of Local Health Departments. COVID-19 School Manual: K–12 public, private, and charter schools. Salt Lake City, UT: Utah Department of Health; 2021. https://coronavirus-download.utah.gov/School/COVID-19_School_Manual_FINAL.pdf

[4] Hershow RB, Wu K, Lewis NM, Low SARS-CoV-2 Transmission in elementary schools—Salt Lake County, Utah, December 3, 2020–January 31, 2021. MMWR Morb Mortal Wkly Rep 2021;70:442–8. 10.15585/mmwr.mm7012e3 33764967 PMC7993560

[5] Varma JK, Thamkittikasem J, Whittemore K, COVID-19 infections among students and staff in New York City public schools. Pediatrics 2021;147:e2021050605. 10.1542/peds.2021-050605 33688033

[6] Volpp KG, Kraut BH, Ghosh S, Neatherlin J. Minimal SARS-CoV-2 transmission after implementation of a comprehensive mitigation strategy at a school—New Jersey, August 20–November 27, 2020. MMWR Morb Mortal Wkly Rep 2021;70:377–81. 10.15585/mmwr.mm7011a2 33735161 PMC7976619

[7] Gettings J, Czarnik M, Morris E, Mask use and ventilation improvements to reduce COVID-19 incidence in elementary schools—Georgia, November 16–December 11, 2020. MMWR Morb Mortal Wkly Rep 2021;70. Epub May 21, 2021. 10.15585/mmwr.mm7021e1 PMC8158891 34043610

[8] Denny TN, Andrews L, Bonsignori M, Implementation of a pooled surveillance testing program for asymptomatic SARS-CoV-2 infections on a college campus—Duke University, Durham, North Carolina, August 2–October 11, 2020. MMWR Morb Mortal Wkly Rep 2020;69:1743–7. 10.15585/mmwr.mm6946e1 33211678 PMC7676642

[9] Prince-Guerra JL, Almendares O, Nolen LD, Evaluation of Abbott BinaxNOW rapid antigen test for SARS-CoV-2 infection at two community-based testing sites—Pima County, Arizona, November 3–17, 2020. MMWR Morb Mortal Wkly Rep 2021;70:100–5. 10.15585/mmwr.mm7003e3 33476316 PMC7821766

[10] Paltiel AD, Zheng A, Walensky RP. Assessment of SARS-CoV-2 screening strategies to permit the safe reopening of college campuses in the United States. JAMA Netw Open 2020;3:e2016818. 10.1001/jamanetworkopen.2020.16818 32735339 PMC7395236

